# Rocky Mountain Spotted Fever, Panama

**DOI:** 10.3201/eid1311.070931

**Published:** 2007-11

**Authors:** Dora Estripeaut, María Gabriela Aramburú, Xavier Sáez-Llorens, Herbert A. Thompson, Gregory A. Dasch, Christopher D. Paddock, Sherif Zaki, Marina E. Eremeeva

**Affiliations:** * Hospital del Niño, Panama City, Panama; † Centers for Disease Control and Prevention, Atlanta, Georgia, USA

**Keywords:** Rickettsia rickettsii, RMSF, Panama, immunohistochemistry, PCR, molecular typing, dispatch

## Abstract

We describe a fatal pediatric case of Rocky Mountain spotted fever in Panama, the first, to our knowledge, since the 1950s. Diagnosis was established by immunohistochemistry, PCR, and isolation of *Rickettsia rickettsii* from postmortem tissues. Molecular typing demonstrated strong relatedness of the isolate to strains of *R. rickettsii* from Central and South America**.**

Rocky Mountain spotted fever (RMSF) is a febrile exanthematic disease caused by *Rickettsia rickettsii*, a gram-negative, obligately intracellular pathogen. *R. rickettsii* primarily invades the endothelium of small and medium-sized blood vessels of all major tissues and organ systems, causing systemic vasculitis. Typical symptoms include headache, fever, rash, and myalgia; meningoencephalitis and renal failure may also occur. The case-fatality rate of untreated RMSF in different areas is 10%–80%.

RMSF is endemic in much of the Western Hemisphere, including Central and South America. In Panama, 5 cases of RMSF, 2 fatal, were reported in the 1950s from the vicinity of Ollas Arriba, Trans-Istmus Highway, and Panama City (*1*–*4*).

In 1952, *R. rickettsii* was isolated from 2 of the patients who died and from a pool of *Amblyomma cajennense* ticks from the Ollas Arriba area, findings that suggest that this tick is a vector of RMSF in Panama (*2*). In 1961, *R. rickettsii* was isolated from a pool of 20 immature *Amblyomma* ticks collected from an opossum and a lizard from the Canal Zone (*5*). Serosurveillance of 1,400 samples tested by complement fixation with *R*. *rickettsii* antigen in 1951–1952 detected 5.4%–15.2% seropositivity in 9 provinces of Panama (*6*). We describe here a recent fatal pediatric case of RMSF, the first report, to our knowledge, of this disease in Panama since the 1950s.

## The Patient

On December 18, 2004, a 4-year-old female patient from a rural area west of the Panama Canal was admitted to Hospital del Niño, Panama City, Panama, with an 8-day history of intense headache, fever, malaise, myalgia, and arthralgia of the lower extremities and 3 days of generalized petechial rash. Ceftriaxone was administered empirically for suspected meningococcemia. Laboratory tests showed hyponatremia (126 mEq/L), hypoalbuminemia (2 mg/dL), thrombocytopenia (48 × 10^9^ cells/L), increased immature neutrophils (26%), and an elevated level of liver enzymes (aspartate aminotransferase 325 U/L and alanine aminotransferase 137 U/L). Results of routine blood cultures were negative. Cerebrospinal fluid analysis showed only protein elevation. Serologic test results for equine encephalitis virus, dengue, hantavirus, and calicivirus as well as bacterial cultures were all negative. On December 18, the patient had seizures, which required intensive care management. Despite intense medical efforts, she died soon thereafter. The main autopsy findings included myocarditis, interstitial nephritis, interstitial pneumonitis, encephalitis, and generalized lymphadenitis. Postmortem frozen unfixed and formalin-fixed tissues were sent to the US Centers for Disease Control and Prevention for etiologic assessment.

**Figure 1:**
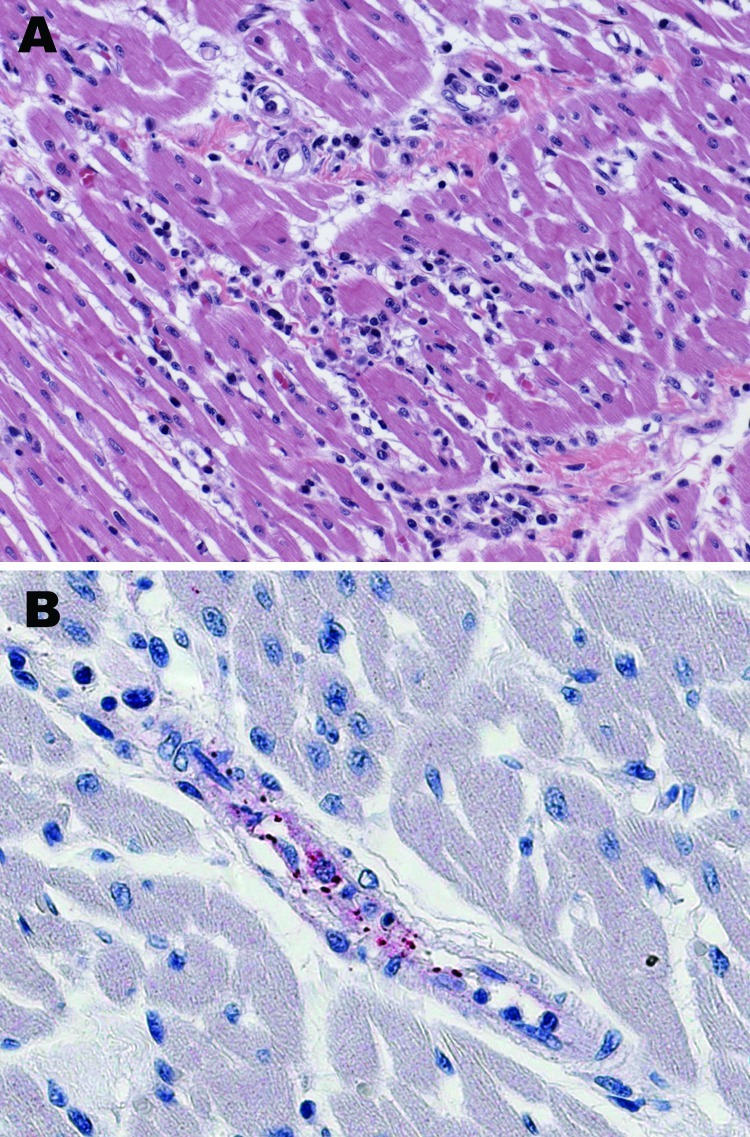
Histologic and immunohistochemical evaluation of heart tissue. A) Lymphohistiocytic inflammatory cell infiltrates in the myocardium (hematoxylin and eosin stain; original magnification ×25). B) Immunohistochemical detection of spotted fever group rickettsiae (red) in perivascular infiltrates of heart (immunoalkaline phosphatase with naphthol-fast red substrate and hematoxylin counterstain; original magnification ×250.

**Figure 2:**
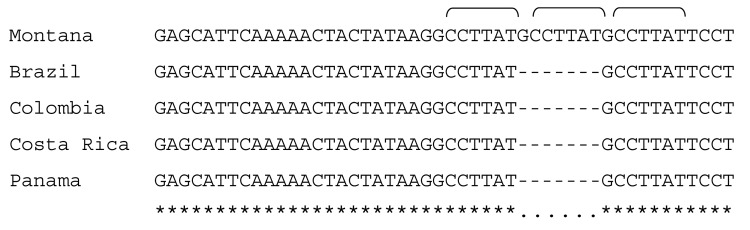
Differentiation of *Rickettsia rickettsii* type strain Sheila Smith from Montana from *R. rickettsii* strains from Central and South America. A tandem repeat region corresponding to 563048–563028 nt of the strain Sheila Smith genome and flanking sequences were amplified with AF (5′-GTGATTGCTATATTTCGCTTT-3′) and AR (5′- CTAAGATTTGTTCCGTATAGG-3′) primers as described elsewhere (*7*). Repeat sequence (GCCTTAT, indicated with brackets) present in 3 copies in strain Sheila Smith, whereas only 2 copies were present in *R. rickettsii* isolates from Brazil, Colombia, Costa Rica, and Panama. Homologous sequences of these strains are deposited to GenBank under the following accession nos.: DQ666020, *R. rickettsii* strain Panama 2004; DQ666021, *R. rickettsii* strain Brazil; DQ666022, *R. rickettsii* strain Colombia; DQ666023, and *R. rickettsii* strain Costa Rica.

Immunohistochemical evaluation of formalin-fixed, paraffin-embedded tissues that used an immunoalkaline phosphatase technique demonstrated spotted fever group rickettsial antigens associated with rickettsia-like cells in vascular endothelium of multiple tissues, including heart (Figure 1), lung, adrenal gland, and kidney. Serum collected on the day of death had immunoglobulin (Ig) G and IgM microimmunofluoresence antibody titers of 2,048 to *R. rickettsii* antigen. DNA samples prepared from frozen brain, liver, lymph node, and spleen autopsy specimens were tested by PCR as described previously (*7*). When amplified, a 208-bp fragment of the conserved 17-kDa *Rickettsia* antigen gene showed spotted fever rickettsial DNA was present in all autopsy tissues. An OmpA gene fragment (70–602 nt) was amplified from brain and lymph node tissues (GenBank accession nos. DQ002503 and DQ002504). DNA sequencing of the *omp*A amplicons demonstrated that their nucleotide sequences were identical to each other and had 100% sequence similarity to the homologous *omp*A fragment of *R. rickettsii* strain Sheila Smith, isolated from a patient in Montana. An isolate of *R. rickettsii* (designated Panama 2004) was obtained from brain tissue in Vero E6 cells; its OmpA gene fragment (DQ164838) had 100% nucleotide sequence similarity to the reference sequence of *R. rickettsii* type strain Sheila Smith. *R*. *rickettsii* from Panama is similar to the other *R. rickettsii* strains circulating in Central and South America but differs from strain Sheila Smith in at least 1 locus containing tandemly repeated sequences (Figure 2).

The clinical characteristics of this patient, e.g., fever, headache, myalgia, petechial rash, and neurologic signs, initially aroused clinical suspicion for a viral fever caused by Venezuelan equine encephalitis (VEE) virus, particularly because there was a VEE outbreak in this area at that time. The failure to diagnose a spotted fever rickettsiosis was partially the result of diminished awareness of RMSF among local physicians and absence of adequate specific diagnostic tests in Panama. Detection of hyponatremia, hypoalbuminemia, and thrombocytopenia, all observed in this patient, indicates endothelial vascular damage and increased vascular permeability and can aid in making a presumptive diagnosis (*8*). Death secondary to RMSF is associated with delays in diagnosis and delays in initiating appropriate antimimicrobial therapy within the first 5 days of the clinical disease (*9*). To prevent fatal outcomes, treatment of suspected case-patients should be initiated before the results of diagnostic tests are received, and treatment should be administered for 7–10 days or until the patient has been without fever for at least 3 days (*9*).

*R. rickettsii* had been isolated previously in Panama from a few patients during the 1950s and from pools of *A. cajennense* ticks during the 1950s and 1960s (*1*,*2*,*5*). As occurred in this instance, cases of RMSF have likely been missed in Panama during the intervening decades; however, the low frequency of recognized cases of RMSF does not correspond to the relatively high seroprevalence of complement-fixing antibodies to spotted fever group rickettsiae in residents of Panama (*6*). Indeed, inoculation of other pools from *Amblyomma* larvae and *A. cajennense* adults caused seroconversion to spotted fever group antigens in guinea pigs, but *R. rickettsii* was not isolated (*5*). Recent studies of *Amblyomma* ticks in Brazil have detected both *R. bellii* and an agent associated with *A. americanum* in the United States known as “*Rickettsia amblyommii*” (*10*). *R. amblyommii* has been implicated as a cause of a mild, self-limiting rickettsial illness in which seroconversion to *R. rickettsii* antigens occurs (*11*,*12*). *R. parkeri*, found in *A. maculatum* and *A. triste,* has been also identified as a cause of disease in the United States and has been presumptively associated with infections of humans in Uruguay (*13*). Since the characterization of rickettsial agents associated with *Amblyomma* species that bite humans is a newly emergent field in the Americas (*13*–*15*), other typhus group and spotted fever group agents may be found in Panama.

## Conclusions

This case confirms that *R. rickettsii* is still present in Panama. A high index of suspicion is necessary for an early diagnosis and empiric treatment of RMSF. Proposed widening of the Panama Canal and current construction of a new portion of the Trans-Istmus Highway disrupts the adjacent forest areas and would likely increase the frequency of human–tick contacts. Thus, RMSF and ehrlichioses should be considered early in the differential diagnosis of febrile infections in tick-exposed persons in Panama. Further study of endemic rickettsioses, rickettsial agents, and possible tick vectors in Panama is warranted.
